# Herb-induced autoimmune-like hepatitis associated with Xiang-tian-guo (Swietenia macrophylla seeds): A case report and literature review

**DOI:** 10.1097/MD.0000000000024045

**Published:** 2021-01-15

**Authors:** Yue-Ming Shao, Yu Zhang, Xin Yin, Ting-Ting Qin, Qing-Long Jin, Xiao-Yu Wen

**Affiliations:** a Department of Hepatology, The First Hospital of Jilin University; b Center for Infectious Diseases, West China Hospital of Sichuan University, Chengdu, China.

**Keywords:** case report, chemical and drug induced liver injury, literature review, xiang-tian-guo

## Abstract

**Rationale:**

Drug-induced liver injury (DILI) has a relatively low incidence, whereas the incidence of herb-induced liver injury (HILI) is still under investigation. As a special type of DILI, the diagnosis of drug-induced autoimmune-like hepatitis presents a persistent challenge, because this condition has partial characteristics of both DILI and autoimmune hepatitis (AIH), such as a certain history of medication use and histology that similar is to AIH. Thus, the differential diagnosis between DILI and AIH can be confusing.

**Patient concerns:**

A 67-year-old woman taking xiang-tian-guo for 6 months was admitted to our hospital with a complaint of experiencing jaundice for 2 weeks.

**Diagnosis:**

A liver biopsy exhibited interface inflammation, foam cells, and “rosette” -like hepatocytes. She was diagnosed with herb-induced liver injury (hepatocellular and acute), a RUCAM score of 7 (probable), a severity for grade 4 liver injury, and accompanied autoimmune-like changes.

**Interventions:**

The patient was instructed to cease the administration of suspicious drugs. The patient also received liver protection and albumin transfusion.

**Outcomes:**

After 25 days of hospitalization, the patients aminotransferase levels returned to normal. No recurrence was observed after the administration of the treatments and a close follow-up.

**Lessons:**

We must to be vigilant about the safety of xiang-tian-guo as a herbal medicine. When faced with the difficulty of distinguishing between AIH and DILI, long-term follow-up observations for recurrence can aid clinicians in making a judgment.

## 1 Introduction

The incidence of DILI is relatively low, and the clinical phenotype is diverse. Additionally, there is a lack of specific biomarkers for DILI, which results in the diagnosis of this disorder to be one of the most challenging diseases that is faced by hepatologists.^[1]^ There are few reports of drug-induced liver injury caused by xiang-tian-guo (Swietenia macrophylla seeds [SMS]). The challenge we faced in our patient was in the identification of AIH. To a certain extent, serology and histology can aid us in diagnosis; however, but when the evidence is insufficient, a long-term follow-up observation can indicate the correct direction of treatment. Therefore, in this report, we reviewed the clinical reports of liver injury caused by xiang-tian-guo in recent years, and we explained the method of how to distinguish between AIH and DILI. Additionally, we suggest that more experiments are needed to verify the human safety of the use of xiang-tian-guo.

## 2 Case presentation

A 67-year-old female was admitted to our department due to the presence of dark urine for 2 weeks and yellow sclera for 5 days. Accompanying symptoms included fatigue and abdominal distension. She had a history of hypertension and had not regularly taken any antihypertensive medication. To control her blood pressure, she reported that she had been taking xiang-tian-guo (Swietenia macrophylla seeds [SMS]) for the past 6 months. She denied having histories of diabetes, blood transfusions, viral hepatitis, or a consumption of alcohol.

A physical examination demonstrated no signs of chronic liver disease except for jaundice. Liver function tests revealed severe changes in the following variables: aspartate aminotransferase (AST) level was 672.4 U/L (normal range: 13–35 U/L), alanine aminotransferase (ALT) level was 681.7 U/L (normal range: 7–40 U/L), glutamyl transpeptidase (γ – GT) level was 161.2 U/L (normal range: 7–45 U/L), alkaline phosphatase (ALP) level was 140.4 U/L (normal range: 50–135 U/L), and total bilirubin (TBL) level was 332.3 μmol/L (normal range: 6.8–30.0 μmol/L). Additionally, her prothrombin time was 18.8 second (normal range: 9.0–13.0 second). The etiologies of hepatitis A, B, C and E were negative. Autoimmune tests revealed only positive indication of the granule type of antinuclear antibodies (ANA). The anti-mitochondrial M2 antibody test was negative. The immunoglobulin tests reported the following results: IgG level at 15.00 g/L (normal range: 7.0–16.0 g/L), IgA level at 4.57 g/L (normal range: 0.7–4.0 g/L), and IgM level at 1.33 g/L (normal range: 0.4–2.3 g/L).Abdominal CT scans indicated liver damage. An ultrasound-guided liver biopsy was also performed.

**Figure 1:**
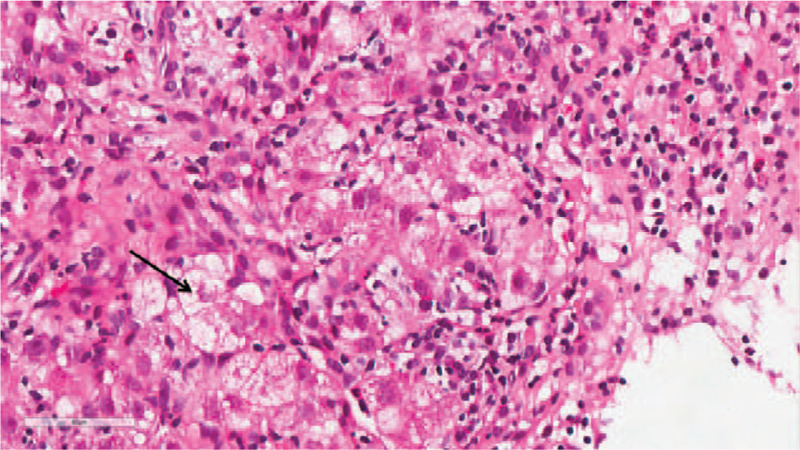
HE staining × 400: The “rosette”- like hyperplasia of hepatocytes (black arrow).

**Figure 2:**
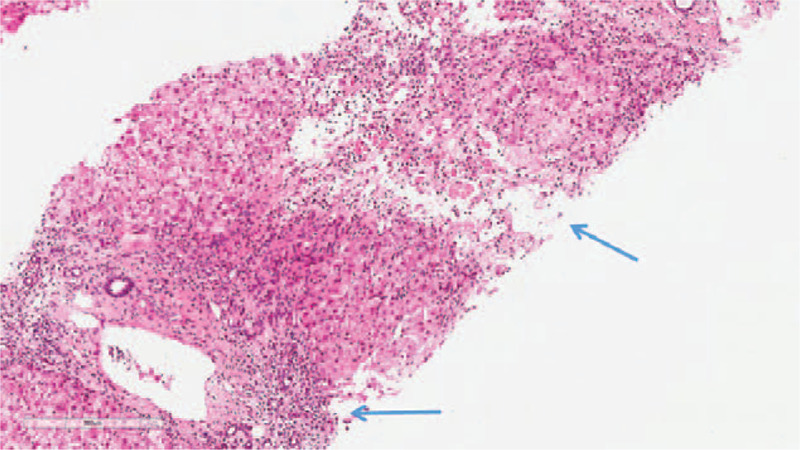
HE staining × 100: Severe focal necrosis and multiple bridging necrosis (blue arrows).

**Figure 3:**
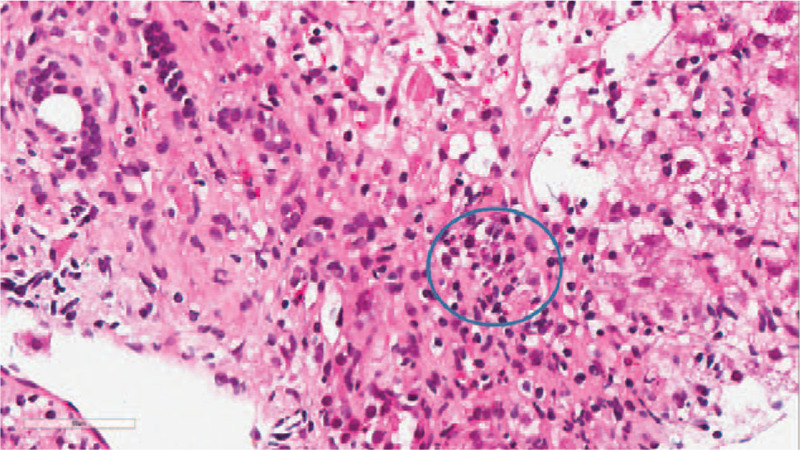
HE staining × 400: Interfacial inflammation with infiltration of foam cells, a little lymphocytes and plasma cells (Inside the circle).

**Figure 4:**
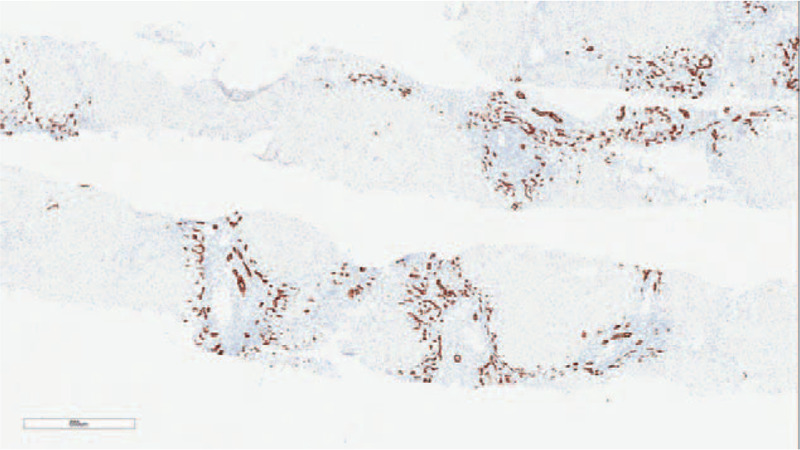
CK7 immunohistochemical staining × 40: There was no evidence of destruction, degeneration and disappearance in the bile duct, while the small bile duct had obvious proliferation.

The liver biopsy revealed that the focal liver acinar structure was abnormal. Specifically, the hepatocytes were diffuse with hydropic degeneration and ballooning degeneration, with a “rosette”- like hyperplasia of the hepatocytes (Fig. 1), which had progressed to a severe focal necrosis and multiple bridging necrosis (Fig. 2). A partial liver plate surrounding the central vein was collapsed, with infiltrations of foam cells, some little lymphocytes, and plasma cells. Kupffer cells had proliferated, and several lymphocytes had infiltrated into the hepatic sinuses. Interfacial inflammation was also observed (Fig. 3). There was no evidence of destruction, degeneration, or disappearance in the bile duct, whereas the small bile duct exhibited obvious proliferation (Fig. 4). Mixed inflammatory cells had also infiltrated into the stroma. The degree of pathological change was equivalent to G4S1 changes. Our pathologist considered that the liver injury may have been caused by drugs or environmental toxoids. The patients were given liver protection and albumin transfusions for 25 days. The liver function gradually recovered, after which the patient was discharged.

The patient was followed up regularly after the discharge. Thus far, her liver function has been normal, and no signs of recurrence were evident. Based on the symptoms, signs, and examination, the clinicians considered that the diagnosis was herb-induced liver injury (hepatocellular and acute), a RUCAM score of 7 (probable), a severity for grade 4 liver injury, and accompanied autoimmune-like changes.

This case report was approved by the ethics committee of the First Hospital of Jilin University, Changchun, China, and the informed consent form was signed by patient.

## 3 Literature review and discussion

Herbal medicines are extensively used for treating various diseases and are universally perceived as being safe, on account of their wide use in traditional Chinese medicine for thousands of years.^[2]^ Currently, with the increasing popularity of herbs, the hepatotoxicity of these drugs has also been realized. For example, the LiverTox and Hepatox databases provide the list of names of the hepatotoxic drugs on the Internet, which helps the public in increasing their knowledge of these drugs. A systematic review^[3]^ conducted in China indicated that, among all cases of DILI, the proportion of traditional Chinese medicine -induced liver injury was 25.71%. In terms of gender and age distributions, women and the elderly account for the majority of the cases in China.^[3,4]^ Common clinical symptoms include jaundice, fatigue, anorexia, and nausea. Similar to the treatment of DILI, treatments involve the cessation of suspicious drugs, the use of hepato-protecting drugs, and the regular monitoring of liver function. Nevertheless, the clinical characteristics of herb-induced liver injury still remains vague.^[5]^

We retrieved 8 reported cases of liver injury related to the use of xiang-tian-guo, for a total of 9 reported cases, including the present case (Table 1). The cases included 4 females and 5 males, with an average age of 64.13 ± 11.73 years. The cases consisted of 2 cases of moderate liver injury (ALT abnormality, 2 × ULN ≤ total bilirubin, or symptomatic hepatitis), 4 cases of severe liver injury (ALT abnormality, 2 × ULN ≤ total bilirubin, with severe symptoms and 1 of the following criteria: international normalized ratio ≥1.5, ascites, and/or encephalopathy, disease duration <26 weeks, and the absence of underlying cirrhosis, failures of other organs due to DILI), and 3 cases that could not be clearly classified. With the exception of 1 case that was evaluated as being a possible case by a pharmacist (according to the WHO adverse drug reaction events evaluation criteria), the remaining 8 patients were assessed for causality by the RUCAM method.^[11]^ Seven cases were probable, and 1 case was evaluated as being high likely, due to positive reactions of reexposure. These 8 cases were clinically classified into the hepatocyte-type of DILI. Jing^[12]^ reported that, when compared to DILI, HILI cases present a higher rate of hepatocellular injury features. A total of 3 patients underwent liver biopsies. One case^[6]^ exhibits bridging necrosis, with inflammation surrounding the portal vein and lobules. The infiltrates were composed of lymphocytes, eosinophils, and plasma cells. Another case.^[7]^ suggested signs of small hepatic vein hemorrhage, with infiltrations of lymphocytes and neutrophils, as well as canalicular and hepatocellular cholestasis. In our case, the histology demonstrated focal necrosis, bridging-like necrosis, “rosette”-like changes in the hepatocytes, interfacial inflammation, and infiltrating cells mainly consisting of foam cells.

**Table 1:** Characteristics of xiang-tian-guo-induced hepatotoxicity cases.

Cases	Sex/Age	Previous illness	AST^∗^ (U/L)	ALT^∗^ (U/L)	ALP^∗^ (IU/L)	TBL^∗^ (umol/L)	RUCAM score	Type of DILI	Liver biopsy	Treatment Period (days)	References
Patient 1	F/45	Depression	1255	1267	124	258	7	Hepatocellular	Y	10	Valerie Yeap, 2018^[6]^
Patient 2	M/72	N/A	789	678	254	212.4	7	Hepatocellular	Y	28	Tan Youwen, 2019^[7]^
Patient 3	M/75	Diabetes	N/A	363	122	N/A	7	Hepatocellular	N	28	Tan Youwen, 2019^[7]^
Patient 4	M/80	Hypertension	N/A	224	108	N/A	7	Hepatocellular	N	14	Tan Youwen, 2019^[7]^
Patient 5	F/66	Diabetes	664	528	168	67.9	9	Hepatocellular	N	27	QiChuanwang, 2019^[8]^
Patient 6	M/62	Diabetes	561.2	765.9	190.7	710.7	8	Hepatocellular	N	22	Zhao Xi, 2019^[9]^
Patient 7	F/61	Diabetes	398	276	138	312.3	8	Hepatocellular	N	33	Zhao Xi, 2019^[9]^
Patient 8	M/52	Diabetes, hypertension	187	521	N/A	N/A	N/A	N/A	N	15	Ding Nan, 2019^[10]^
Patient 9	F/67	Hypertension	672.4	681.7	140.4	332.3	7	Hepatocellular	Y	25	Our case

∗ The laboratory test results in the table are at the time of admission. ALP = alkaline phosphatase, ALT = alanine aminotrans ferase, AST = aspartate aminotrans ferase, DILI = drug-induced liver injury, F = Female, M = Male, N = No, N/A = not applicable, TBL = total bilirubin, Y = Yes.

We observed that interfacial inflammation, lymphocytic/plasmocytic infiltration in the portal area, and the “rosette”-like structures in the hepatocytes are the histological features for AIH. However, a retrospective study in recent years demonstrated that the “rosette”-like structures in the patients with drug-induced, autoimmune-like hepatitis are more common than those in patients with drug-induced liver injury without evidence of autoimmunologic hepatitis, but the difference was not statistically significant.^[12]^ A recent study also demonstrated that “rosette”-like changes are difficult to define and are difficult to diagnose based on the simplified criteria^[13]^ DILI with autoimmune characteristics cannot be reliably distinguished from AIH from the sole perspective of histology.^[14]^ Another recently published retrospective study also demonstrated that 243 liver biopsy-proven HILI cases were immune-mediated, which accounted for 53.9% of the total cases.^[15]^ Manuel^[16]^ reported a case of hyperammonemia that was caused by Olmesartan. The patients pathological characteristics were similar to AIH, but no immunosuppressants were used. Only the discontinuation of the suspected drug, hypertransaminasemia was recovered. The researchers considered that the drug-induced liver injury may be mediated by an immune mechanism. According to the EASL guidelines,^[1]^ for the patients with initial onsets, a clear medication history, and obvious autoimmune characteristics that cannot be diagnosed, even after the discontinuation of the suspected drugs, immunosuppressive therapy can be considered, and this therapy can be gradually reduced the amount until the drug is stopped. If there is no sign of recurrence during the follow-up process, then the DILI diagnosis can be established. If there is no indication of remedication and the disease recurs, then it can be diagnosed as AIH.^[17]^ Our patients serum IgG level was normal. Additionally, only granule types of cells were positive in the ANA series. A widely accepted study demonstrated that, although nonspecific, ANA results are positive in 50% to 83% patients with autoimmune-like, drug-induced liver injury.^[18–20]^ Another case report published in Hepatology also demonstrated that the ANA series can be positive for immune-mediated, drug-induced liver injury.^[21]^ No immunosuppressive therapy was used. The liver function was normal within 6 months after discharge; therefore, the evidence of the diagnosis of AIH was insufficient. At the same time, the guidelines indicates that individuals carrying the HLA allele DRB1 ∗ 03:01 / ∗ 04:01 can be more easily diagnosed with idiopathic AIH, and the presence of the DILI risk allele DRB1∗15:01 supports the diagnosis of drug-induced AIH. The limitation of this case was that we did not assess for the presence of this gene, which may be related to the identification of the AIH and DILI, as these tests were not available in our hospital. Therefore, our patient was diagnosed with herb-induced liver injury and accompanied autoimmune-like changes. This is the first case of herb-induced, autoimmune-like hepatitis caused by xiang-tian-guo that was reported in China.

Xiang-tian-guo belongs to the mahogany genus Meliaceae. It is known as the Swietenia macrophylla seeds and is also called the Tunjuk Langit in Malaysia and skyfruit in the English language. The seeds are used to treat diabetes and hypertension. In 2015, Balijepalli^[22]^ evaluated the safety of SMS in Sprague-Dawley (SD) rats. Their conclusion was that the rat dose of 2 g/kg bw, which is equivalent to the human dose of 325 mg/kg bw and well below the common amount that is consumed by people, did not exhibits any signs of toxicity in rats. Chng YS^[23]^ demonstrated that SM50 has the potential to be used as an herbal medication to treat hypertension. However, there have been no further clinical trials of this herb to confirm its safety. We collected clinically relevant case reports, and all of these cases exhibited good prognoses after treatment. We envision further research on this phenomenon, and we hope to alert clinicians of this drug's potential liver toxicity.

## Acknowledgment

We appreciate the histological images provided by Jin Meishan from the Department of Pathology, the first hospital of Jilin university.

## Author contributions

**Conceptualization:** Yue-ming Shao, Yu Zhang, Xin Yin, Xiao-yu Wen.

**Data curation:** Yue-ming Shao.

**Funding acquisition:** Xiao-yu Wen.

**Investigation:** Yue-ming Shao, Yu Zhang.

**Resources:** Yu Zhang, Xin Yin, Ting-ting Qin.

**Supervision:** Qing-long Jin.

**Writing – original draft:** Yue-ming Shao.

**Writing – review & editing:** Xiao-yu Wen.

## Abbreviations

Abbreviations: AIH = autoimmune hepatitis, ALP = alkaline phosphatase, ALT = alanine aminotransferase, ANA = antinuclear antibodies, AST = aspartate aminotransferase, DILI = drug-induced liver injury, HILI = herb-induced liver injury, SMS = Swietenia macrophylla seeds, TBL = total bilirubin, ULN = upper limit of normal.
